# Molecular Insights Into the Phylogenetic Diversity, Virulence Determinants, and Antimicrobial Resistance Pattern of *Escherichia coli* From Milk of Mastitis‐Affected Cows

**DOI:** 10.1155/vmi/3499907

**Published:** 2026-08-20

**Authors:** Md. Mominul Islam, Ayesha Akter, Md. Sadequl Islam, Mahfuzul Islam, S. M. Harun ur Rashid, Mirza Mienur Meher, Jahagir Alam

**Affiliations:** ^1^ Department of Pathology and Parasitology, Hajee Mohammad Danesh Science and Technology University, Dinajpur 5200, Bangladesh, hstu.ac.bd; ^2^ Department of Pathology, Sher-e-Bangla Agricultural University, Sher-e-Bangla Nagar, Dhaka 1207, Bangladesh, sau.edu.bd; ^3^ Department of Anatomy and Histology, Hajee Mohammad Danesh Science and Technology University, Dinajpur 5200, Bangladesh, hstu.ac.bd; ^4^ Department of Microbiology and Parasitology, Sher-e-Bangla Agricultural University, Sher-e-Bangla Nagar, Dhaka 1207, Bangladesh, sau.edu.bd; ^5^ Department of Microbiology and Public Health, Faculty of Veterinary Medicine and Animal Science, Gazipur Agricultural University, Gazipur 1706, Bangladesh; ^6^ Department of Anatomy, Histology and Physiology, Sher-e-Bangla Agricultural University, Sher-e-Bangla Nagar, Dhaka 1207, Bangladesh, sau.edu.bd

**Keywords:** antimicrobial resistance, bovine mastitis, *E. coli*, phylogeny, resistance genes

## Abstract

This study examined the prevalence, molecular characterization, and antimicrobial resistance of *Escherichia coli* associated with the bovine clinical mastitis in the northern part of Bangladesh. A total of 120 milk samples from infected cows in Dinajpur, Thakurgaon, and Nilphamari were collected. General bacteriological and biochemical tests were conducted to isolate and identify the bacteria. In addition, PCR assays were performed to molecularly detect and identify virulence and antibiotic resistance genes. Bacteriological, biochemical, and molecular methods confirmed the presence of *E. coli*, which constituted a total prevalence of 35.0%. Phylogenetic typing revealed two types, A1 (81.0%), and B2 (19.0%) (*p* < 0.001), suggesting that commensal strains predominating the total *E. coli* population. Virulence gene (*eae, stx1,* and *stx2*) screening was negative, indicating a lack of classical enteropathogenic/enterohemorrhagic characteristics. Antibiotic susceptibility testing showed that the level of antibiotic resistance was high, especially to ampicillin (100.0%), amoxicillin (92.86%), tetracycline (73.81%), erythromycin (61.91%), streptomycin (59.53%), and gentamicin (52.38%). The isolates were mostly sensitive to ceftriaxone (85.71%) and moderately sensitive to chloramphenicol and ciprofloxacin. The most abundant resistance gene was *tet*A (85.71%), followed by sul2 (59.52%), *blaTEM* (57.14%), *aadA1* (50.0%), *qnr* (23.80%), and *blaCTX-M* (19.04%) (*p* < 0.001), which are strongly connected to the phenotypic resistance pattern. In general, the results indicate that highly resistant *E. coli* are prevalent in the case of bovine mastitis, which highlights the necessity to use antibiotics reasonably, conduct more frequent monitoring, and use biosecurity to limit the spread of resistant pathogens in the dairy industry, as well as the environment.

## 1. Introduction

Bovine mastitis, known as the inflammation of the mammary gland, is one of the most important infectious diseases in dairy cows all over the world. It does not only lead to massive economic losses in terms of reduced milk production, milk dumping, veterinary services, and untimely culling but also has significant social health implications with regard to the possibility of transfer of zoonotic pathogens and antibiotic residues through milk [1–3]. The disease is classified into clinical and subclinical types, and the clinical form is easier to detect because of the presence of such manifestations as swelling, erythema, pain, and a shift in milk consistency. In contrast, subclinical mastitis is insidious and cannot be detected without diagnostic screening; nevertheless, it leads to losses in long‐term production [4, 5].

Various types of bacteria may lead to mastitis; however, *Escherichia coli* (*E. coli*) is one of the most commonly encountered forms of mastitis in clinical practice [4]. *E. coli* is a Gram‐negative, facultative anaerobic bacillus that is a normal flora of bovine intestine and may invade the mammary gland when the environment is unsanitary or immunocompromised, leading to inflammatory reactions in the body and systemic disease [4–6]. Its capacity to colonize the mammary gland has been associated with various virulence factors including adhesins, siderophores, and lipopolysaccharides (LPS), which are pathogenicity factors and evasion of immunity [7]. The role of *E. coli* in mastitis is also becoming more noted in intensive and smallholder dairy production, especially in low‐ and middle‐income nations where hygiene and disease surveillance are not necessarily optimal [8]. The management of bovine mastitis in the last 20 years has widely used the application of broad‐spectrum antibiotics. Nevertheless, the indiscriminate use of antimicrobial agents in the dairy industry has led to the alarming increase in antibiotic resistance in mastitis agents, particularly *E. coli* [9, 10]. Multidrug‐resistant (MDR) strains of *E. coli* have emerged in both clinical and environmental specimens, and this raises questions regarding therapeutic problems and threats to human health because of horizontal gene transfer and antimicrobial drug residues in milk [11]. MDR *E. coli* was defined as a strain showing nonsusceptibility to at least one agent in three or more antimicrobial categories [12]. The emergence of resistance against prevalent antibiotics like ampicillin, tetracycline, and beta‐lactams has become an issue of concern, especially where the development of resistance is orchestrated by the presence of particular resistance genes, namely, *blaTEM*, *blaCTX-M*, *TetA*, and *aadA1* [13, 14].

Genotyping of *E. coli* isolates in cases of mastitis offers fundamental data regarding the diversity of strains, their virulence, and their evolutionary background. There are some genotypes that are more likely to cause an illness as they harbor certain virulence factors, while others can be less harmful or even normal bacteria in the flora [15]. Thus, the phylogenetic classification provides an opportunity to group *E. coli* into particular genotypes, which can be linked to the variation of the pathogenic profile and resistance pattern [15]. As an example, A1 can be regarded as commensal, but it has been implicated with opportunistic infections increasingly [16], whereas B2 and other B‐group genotypes have been linked to increased ability to become virulent [17–19]. It is thus important that molecular characterization is relevant to the process of epidemiological trends, and is able to influence effective treatment and control strategies.

Mastitis has been a big problem in smallholder and commercial dairy production in Bangladesh [20, 21]. Although the dairy industry is expanding in the country, a little is known about the molecular epidemiology of mastitis causative agents, especially *E. coli*. This knowledge gap is very serious because dairy cows are often subjected to contaminated water, improper milking systems, and the uncontrolled use of antibiotics, which in turn might encourage the development, and transmission of resistant pathogens [20]. Some earlier studies have given attention to the bacteriological profile of mastitis; however, detailed studies taking into account phenotypic and genotypic together with resistance profiling of *E. coli* are yet to be undertaken in the Bangladesh setup [22, 23]. To fill this research gap, the current study was carried out in three dairy‐rich northern districts of Bangladesh, namely, Thakurgaon, Nilphamari, and Dinajpur. These areas are high‐production areas of milk where mastitis is widely reported but has not been well established in terms of recording the extent of occurrence at the molecular level. The objective of the study was to isolate and identify *E. coli* in the mastitis‐infected cows, characterize their genotypes, determine the antibiotic resistance phenotype through disc diffusion methods, and identify the important resistance genes.

## 2. Materials and Methods

### 2.1. Ethical Considerations

Ethical waiver was obtained from the ethical committee of Hajee Mohammad Danesh Science and Technology University as the study involved only noninvasive milk sampling (No. 1812/2022). In addition, written consent was obtained from all dairy farm owners prior to sample collection.

### 2.2. Sample Collection

A total of 120 milk samples were collected from dairy cows in three northern districts of Bangladesh: Dinajpur, Nilphamari, and Thakurgaon. The samples were collected from 45 small holder dairy farms, containing 10–30 dairy cows. The study population consisted of lactating dairy cows (crossbred and local breeds) exhibiting clinical signs of mastitis and rearing under intensive and semi‐intensive management systems. The occurrence of mastitis was assessed by the veterinarian. Only one sample was collected from the cows with clinical mastitis, and samples were collected before the start of antibiotic treatment. Milk was aseptically drawn from quarters displaying visible symptoms such as udder swelling, tenderness, fever, and signs of systemic illness, including reduced appetite and lethargy. Approximately 10 mL of milk was collected into sterile 15 mL Falcon tubes and transported in an ice box with ice to the laboratory. Samples were stored at 4°C and processed within 24 h of collection.

### 2.3. Isolation and Identification of *E. coli*

Enrichment and isolation of *E. coli* were performed following the method of Bag et al. [24], with minor modifications. Briefly, 500 μL of each milk sample was inoculated into 4.5 mL of Luria‐Bertani (LB) broth (HiMedia, India) and incubated overnight at 37°C. From the enriched culture, a loop full of enriched sample was streaked onto Eosin Methylene Blue (EMB) agar (HiMedia, India) and incubated at 37°C for 18–24 h. Colonies showing a greenish metallic sheen were considered presumptive *E. coli* and were subcultured for purification. Isolates were further confirmed by Gram staining and a series of biochemical tests, including catalase, oxidase, indole production, methyl red, Voges–Proskauer (VP), and citrate utilization tests.

### 2.4. Molecular Confirmation of *E. coli*

Genomic DNA was extracted from freshly cultured colonies using the Qiagen DNA Mini Kit (Qiagen, Germany), following the manufacturer’s instructions. Specific detection of *E. coli* was performed via PCR as described by Riffon et al. [25]. Each 20 μL reaction mixture included 10 μL of 2X GoTaq G2 Green Master Mix (Promega, USA), 10 pmol of each forward and reverse primer, and 3 μL of DNA template. DNA from *E. coli* served as a positive control. Thermal cycling was performed in a thermal cycler with the following conditions: initial denaturation at 95°C for 3 min; 30 cycles of 94°C for 45 s, 58°C for 45 s, and 72°C for 60 s, followed by a final extension at 72°C for 3 min (Table 1).

**TABLE 1:** Primers used in this study.

SL no.	Primer name	Sequences (5′‐3′)	Amplicon size (bp)	PCR amplifications (35 cycles)					References
				ID °C (min.)	DEN °C (sec.)	ANL °C (sec)	EXT °C (sec)	FEXT °C (min)	
1	Eco 223	ATC AAC CGA GAT TCC CCC AGT	232	95/3	94/45	58/45	72/60	72/3	[25]
	Eco 455	TCA CTA TCG GTC AGT CAG GAG							

2	ChuA.1	GACGAACCAACGGTCAGGAT	279	94/4	94/30	59/30	72/30	72/5	[26]
	ChuA.2	TGCCGCCAGTACCAAAGACA							
3	YjaA.1	TGAAGTGTCAGGAGACGCTG	211						
	YjaA.2	ATGGAGAATGCGTTCCTCAAC							
4	TspE4C2.1	GAGTAATGTCGGGGCATTCA	152						
	TspE4C2.2	CGCGCCAACAAAGTATTACG							

5	Eae1	F: AAACAGGTGAAACTGTTGCC	490	95/3	95/40	55/60	72/60	72/10	[27]
	Eae2	R: CTCTGCAGATTAACCTCTGC							

6	*Stx1*‐F	ATCAGTCGTCACTCACTGGT	110	95/3	95/40	57/40	72/30	72/8	[28]
	*Stx1*‐R	CTGCTGCTGTCACAGTGACAA							

7	*Stx2*‐F	CAACACTGGATGATCTCAG	350	95/3	95/40	55/60	72/60	72/10	[29]
	*Stx2*‐R	CCCCCTCAACTGCTAATA							

8	Tet (A)‐F	GGTTCACTCGAAC GACGTCA	577	94/5	94/30	50/30	72/60	72/10	[30]
	Tet (A)‐R	CTGTCCGACAAGT TGCATGA							

9	Tet (B)‐F	CCTCAGCTTCTCAACGCGTG	634	94/5	94/30	50/30	72/60	72/10	[29]
	Tet (B)‐R	GCACCTTGCTGATGACTCTT							

10	aadA1‐F	TATCCAGCTAAGCGCGAACT	447	95/15	94/30	58/30	72/60	72/10	[29]
	aadA1‐R	ATTTGCCGACTACCTTGGTC							

11	aac [3]‐IV‐F	CTTCAGGATGGCAAGTTGGT	286	95/15	94/30	58/30	72/60	72/10	[29]
	aac [3]‐IV‐R	TCATCTCGTTCTCCGCTCAT							

12	ereA‐F	GCCGGTGCTCATGAACTTGAG	419	95/15	94/30	58/30	72/60	72/10	[29]
	ereA‐R	CGACTCTATTCGATCAGAGGC							

13	Sul2‐F	CGGCATCGTCAACATAACCT	721	95/15	94/30	58/30	72/60	72/10	[31]
	Sul2‐R	TGTGCGGATGAAGTCAGCTC							

14	qnr R	GGGTATGGATATTATTGATAAAG	662	95/5	94/30	57/30	72/60	72/5	[32]
	qnr F	CTAATCCGGCAGCACTATTA							

15	blaTEM F	TTTCGTGTCGCCCTTATTCC	692	94/5	94/30	58/30	72/90	72/5	[33]
	blaTEM R	CCGGCTCCAGATTTATCAGC							

16	blaSHV F	GGGTTATTCTTATTTGTCGC	567	94/10	94/60	58/60	72/60	72/10	[34]
	blaSHV R	TTAGCGTTGCCAGTGCTC							

17	blaCTXM F	CCAGAATAAGGAATCCCATG	964	94/5	94/30	51/30	72/60	72/10	[35]
	blaCTXM R	GCCGTCTAAGGCGATAAAC							

*Note:* DEN = denaturation, ANL = annealing temperature, EXT = extension, FEXT = final extension.

Abbreviation: ID = initial denaturation.

### 2.5. Antimicrobial Susceptibility Testing

The antibiotic susceptibility of the confirmed *E. coli* isolates was assessed by the disk diffusion method, according to the Clinical and Laboratory Standards Institute guidelines, 2022 [36]. The plates were incubated at 35°C for 18 h. Results were interpreted as susceptible, intermediate, or resistant. A total of 14 antimicrobial agents representing eight different classes commonly used in both veterinary and human medicine in Bangladesh were tested using commercially available discs (Oxoid, UK): ampicillin (AMP) (10 μg), amoxicillin (AMX) (10 μg), ciprofloxacin (CIP) (5 μg), chloramphenicol (CHL) (30 μg), streptomycin (STR) (30 μg), gentamicin (GEN) (10 μg), erythromycin (ERY) (15 μg), tetracycline (TET) (30 μg), ceftazidime (CFT) (30 μg), ceftriaxone (CTR) (30 μg), azithromycin (AZM) (15 μg), kanamycin (KAN) (30 μg), amikacin (AMK) (30 μg), and cephalexin (CPL) (30 μg).

### 2.6. Genotyping of *E. coli* Isolates

Phylogenetic grouping of *E. coli* isolates was conducted using triplex PCR, following the method described by Carlos et al. [26]. This assay differentiates strains into phylogroups A, B1, B2, and D. The PCR was prepared in a 20 μL volume containing 10 μL of 2X GoTaq Green Master Mix (Promega, USA), 1 μL of each primer (forward and reverse), 2 μL of genomic DNA, and 6 μL of nuclease‐free water. The cycling protocol was as follows: initial denaturation at 94°C for 5 min; 30 cycles of 94°C for 30 s, 55°C for 30 s, and 72°C for 30 s; and a final extension at 72°C for 7 min. PCR products were visualized on 1.2% agarose gels stained with ethidium bromide (0.5 μg/mL) and imaged under UV transillumination. Band sizes were compared against a 100 bp DNA ladder to determine the corresponding phylogroups.

### 2.7. Detection of Virulence and Antimicrobial Resistance Genes

The presence of virulence and antimicrobial resistance genes was assessed in selected *E. coli* isolates using conventional PCR. Based on the phenotypic resistance patterns, the following genes were targeted for detection of *eae, stx1, stx2*, *tetA*, *aadA1*, aac(3)‐IV, *ereA*, *Sul2*, *blaTEM*, *blaSHV*, and *blaCTX-M* genes. PCRs were set up in 20‐μL volumes using the same reagent mix and concentrations as previously described. Primer sequences and PCR conditions are listed in Table 1. PCR products were resolved by agarose gel electrophoresis and visualized under UV light.

### 2.8. Statistical Analysis

The data obtained from this study were analyzed using Minitab 17 program (Minitab Ltd., UK). Significant difference among the variables was calculated using Pearson’s chi‐square test. The statistical significance was declared at *p* < 0.05.

## 3. Results

### 3.1. Prevalence of *E. coli* in Clinical Mastitis Milk Samples

Out of 120 clinical mastitis milk samples examined, 42 were confirmed positive for *E. coli*, yielding an overall prevalence of 35.0% (Table 2). The isolated bacteria showed typical growth patterns on different culture media. Most notably, on EMB Agar, colonies showed a greenish metallic sheen, a hallmark of *E. coli* presence due to acid production from lactose fermentation. All isolates tested positive for indole production, methyl red, and motility, while negative for citrate utilization, oxidase, and VP reaction. On Triple Sugar Iron (TSI) medium, all isolates produced acid in both slant and butt (A/A) with gas production but no hydrogen sulfide (H_2_S), which is characteristic of *E. coli*. Regional variations in prevalence were observed, however not significant (*p* = 0.909). The highest prevalence was noted in Thakurgaon (38.46%), followed by Dinajpur (34.61%) and Nilphamari (33.33%), These findings suggest that *E. coli* is a significant mastitis‐causing pathogen in these districts, though its distribution may vary between localities due to differences in hygiene, farm management, or environmental exposure.

**TABLE 2:** Prevalence of *E. coli* isolated from bovine clinical mastitis.

Regions	No. of samples	No. of positive samples	Prevalence (%)	Chi‐square *p* value
Dinajpur	52	18	34.61	0.909
Nilphamari	42	14	33.33	
Thakurgaon	26	10	38.46	

Total	120	42	35.0	

### 3.2. Molecular Confirmation of *E. coli*

**FIGURE 1:**
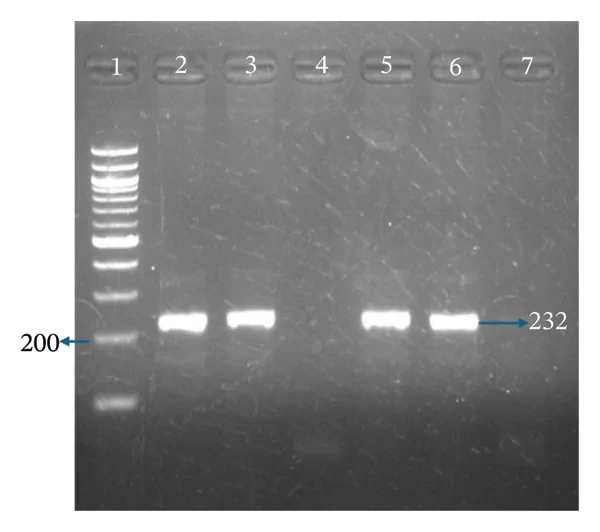
PCR amplification of *E. coli*. Lane 1 contains the DNA ladder, and lane 2 contains the positive control DNA. Lanes 3–6 contain different samples, while lane 7 contains the negative control. The *E. coli*–specific amplicon is observed at 232 bp. A 100‐bp DNA ladder was used as the molecular size marker.

*E. coli* isolates were further confirmed by PCR targeting the 16S rRNA gene. A distinct DNA band of 232 bp was observed in PCR assay. Positive control and negative control validated the assay (Figure 1). The molecular approach provided robust confirmation of the biochemical findings.

### 3.3. Phylogenetic Genotyping of *E. coli*

**FIGURE 2:**
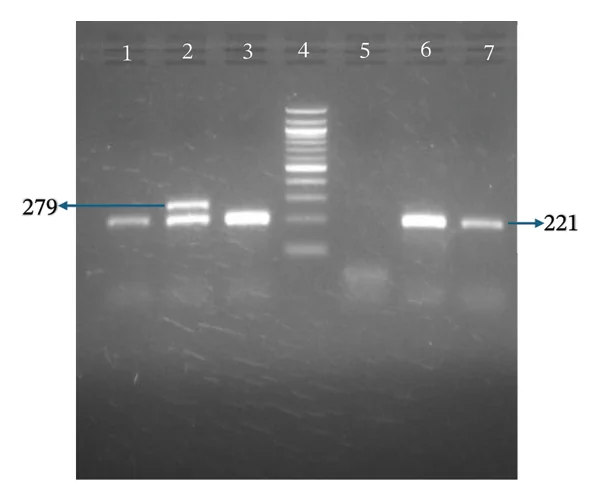
Genotyping of *E. coli*. Lane 4 contains the DNA ladder, while lanes 1–3 and 5–7 contain different samples. The *chuA* gene amplicon is observed at 279 bp, and the *yjaA* gene amplicon is observed at 211 bp. A 100 bp DNA ladder was used as the molecular size marker.

Phylogenetic analysis using triplex PCR identified two predominant genotypes among the isolates. Genotype A1 was the most common, found in 34 of the 42 isolates (81.0%), while Genotype B2 was detected in the remaining 8 isolates (19.0%). Other genotypes such as B1 and D were not observed (Table 3). This result suggests that A1‐type strains, typically commensal or less virulent, are the dominant *E. coli* phylogroup in this region (Table 3, Figure 2).

**TABLE 3:** Phylogenic groups of *E. coli*.

Phylogenetic group	Positive number	Percentage (%)	Indication (27)
A	34	81	chuA−, TspE4.C2‐
B1	0		chuA−, TspE4.C2+
B2	08	19	chuA+, yjaA+
D	0		chuA+, yjaA−

Chi‐square *p* value	< 0.001		

### 3.4. Virulence Gene Detection

None of the *E. coli* isolates tested positive for the virulence genes *eae*, *stx1*, or *stx2*. These genes are commonly associated with enteropathogenic and enterohemorrhagic *E. coli*, suggesting that the isolates in this study may be nonenteric and possess lower pathogenic potential in terms of zoonotic transmission.

### 3.5. Antibiotic Susceptibility Profile

**FIGURE 3:**
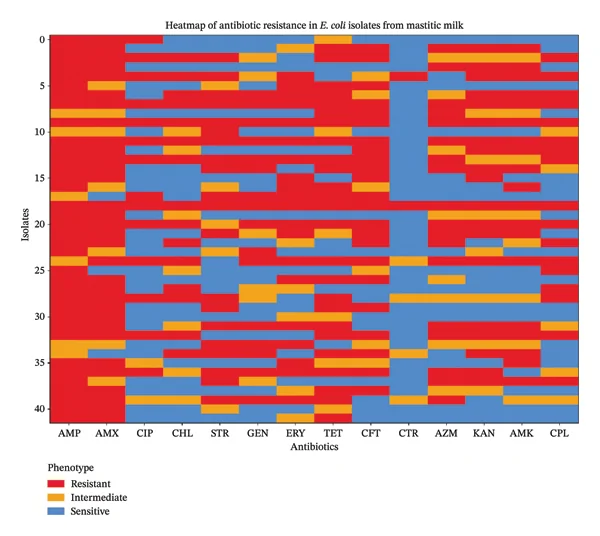
Heatmap showing the antibiotic resistance patterns of *E. coli* isolates recovered from the milk of mastitis‐affected cows. Sensitive isolates are represented in blue, intermediate isolates in orange, and resistant isolates in red. AMP = ampicillin; AMX = amoxicillin; CIP = ciprofloxacin; CHL = chloramphenicol; STR = streptomycin; GEN = gentamicin; ERY = erythromycin; TET = tetracycline; CFT = cefotaxime; CTR = ceftriaxone; AZM = azithromycin; KN = kanamycin; AMK = amikacin; and CPL = cephalexin.

The antimicrobial susceptibility patterns established through the use of 42 *E. coli* isolates obtained from mastitic milk samples were tested against the 14 commonly used antibiotics. According to the heatmap (Figure 3), the level of resistance between isolates and between antibiotics is different. All in all, the isolates had high multidrug resistance. The greatest resistance was detected with ampicillin and amoxicillin, in which almost all of the isolates (100% and 92.86%, respectively) were resistant. There was also significant resistance to tetracycline (73.81%), erythromycin (61.91%), gentamicin (52.38%), and streptomycin (59.53%). Moderate resistance to chloramphenicol (52.38%), ciprofloxacin (38.10%), and ceftazidime (69.09%) was observed. Conversely, the isolates were mostly susceptible to ceftriaxone, which had the highest rate of sensitivity (85.71%), followed by chloramphenicol (47.62) and ciprofloxacin (61.90). Some intermediary responses were also found, especially to amoxicillin, gentamicin, and tetracycline, which were manifested by orange in the heatmap.

### 3.6. Detection of Antibiotic Resistance Genes

**FIGURE 4:**
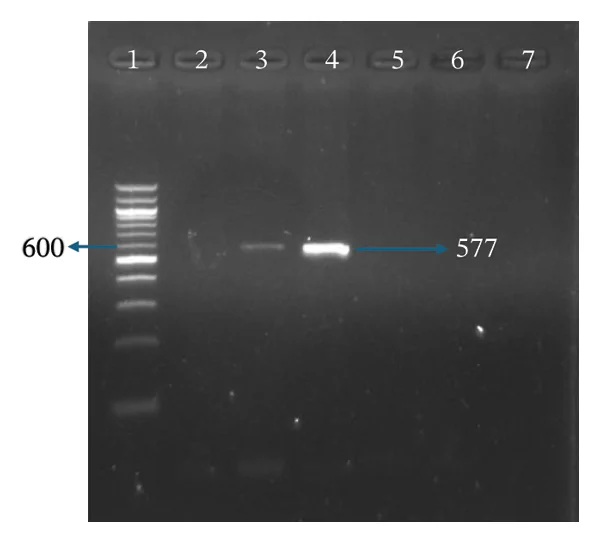
PCR amplification of the antibiotic resistance gene *tetA* in *E. coli.* Lane 1 contains the DNA ladder, lanes 2–6 contain different samples, and lane 7 contains the negative control (NC). The *tetA* amplicon is observed at 577 bp. A 100‐bp DNA ladder was used as the molecular size marker.

**FIGURE 5:**
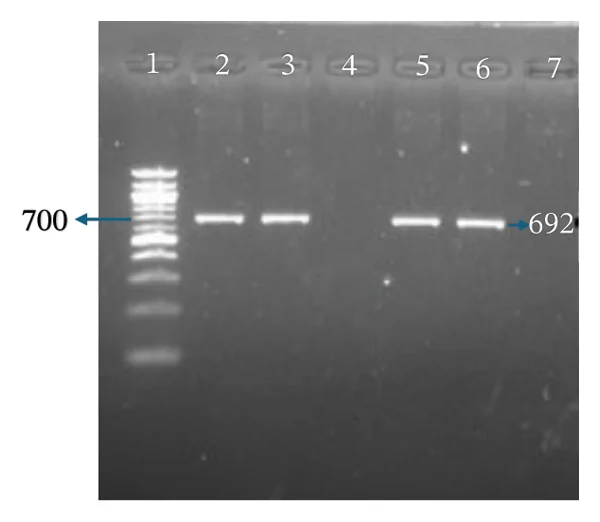
PCR amplification of the antibiotic resistance gene *blaTEM* in *E. coli.* Lane 1 contains the DNA ladder, lanes 2–6 contain different samples, and lane 7 contains the negative control (NC). The *blaTEM* amplicon is observed at 692 bp. A 100‐bp DNA ladder was used as the molecular size marker.

**FIGURE 6:**
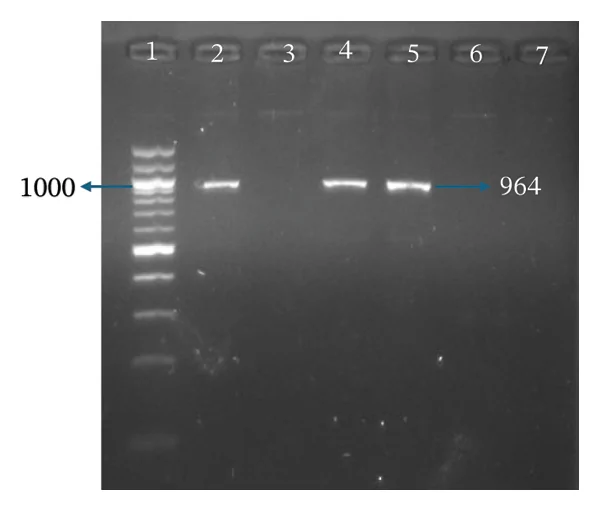
PCR amplification of the antibiotic resistance gene *blaCTX-M* in *E. coli.* Lane 1 contains the DNA ladder, lanes 2–6 contain different samples, and lane 7 contains the negative control (NC). The *blaCTX-M* amplicon is observed at 964 bp. A 100‐bp DNA ladder was used as the molecular size marker.

**FIGURE 7:**
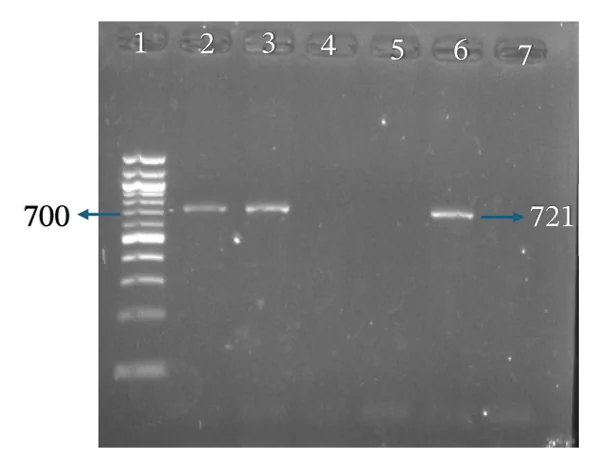
PCR amplification of the antibiotic resistance gene *sul2* in *E. coli.* Lane 1 contains the DNA ladder, lanes 2–6 contain different samples, and lane 7 contains the negative control (NC). The *sul2* amplicon is observed at 721 bp. A 100‐bp DNA ladder was used as the molecular size marker.

**FIGURE 8:**
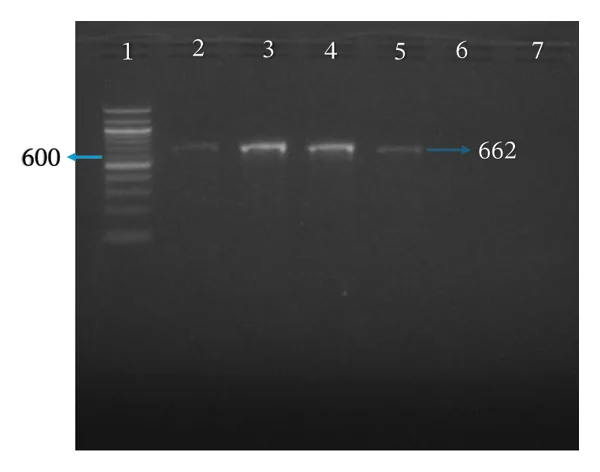
PCR amplification of the antibiotic resistance gene *qnr* in *E. coli.* Lane 1 contains the DNA ladder, lanes 2–6 contain different samples, and lane 7 contains the negative control (NC). The *qnr* amplicon is observed at 662 bp. A 100‐bp DNA ladder was used as the molecular size marker.

PCR‐based screening revealed the genetic determinants underlying the observed phenotypic resistance (Table 4). The *tetA* gene, associated with tetracycline resistance, was detected in 85.71% of isolates (Figure 4), while *blaTEM*, conferring β‐lactam resistance, was found in 57.14% (Figure 5). *blaCTX*‐M, another β‐lactamase gene linked to extended‐spectrum β‐lactam resistance, was present in 19.04% of isolates (Figure 6). In addition, *aadA1*, associated with streptomycin resistance, was identified in 50.0% of isolates. Two other antibiotic resistance genes, *sul2* for sulfonamide and *qnr* for quinolones, were also detected as 59.52% and 23.80%, respectively (Figures 7 and 8).

**TABLE 4:** Antibiotic resistance genes detection of *E. coli*.

Antibiotic resistance gene	Resistance for antibiotics	No. of sample tested	Positive number	Percent (%)	Chi‐square *p* value
*TetA*	Tetracycline	42	36	85.71	< 0.001
*aadA1*	Streptomycin		21	50	
*blaTEM*	Betalactum		24	57.14	
*blaCTXM*	Betalactum		8	19.04	
*Sul2*	Sulphonamides		25	59.52	
*qnr*	Quinolones		10	23.80	

## 4. Discussion

This study investigated the prevalence, genotypic diversity, and antibiotic resistance profile of *E. coli* isolated from mastitis‐affected dairy cows in three northern districts of Bangladesh. The findings contribute valuable insights into the local epidemiology of *E. coli* in bovine mastitis, a disease of major concern in both veterinary and public health contexts. The overall prevalence of *E. coli* among mastitic milk samples was 35%, with slightly higher detection rates in Thakurgaon (38.46%) and Dinajpur (34.61%). These findings are consistent with previous reports from other regions of Bangladesh and South Asia, where prevalence has ranged between 20% and 40% depending on geographic, seasonal, and hygienic conditions [21, 33, 37].

The resistance to ampicillin is nearly universal, and the resistance to amoxicillin is very high, which is also in line with other South Asian studies, in which beta‐lactam resistance is often described as the dominant one in mastitis isolates [14, 24]. We have also found tetracycline resistance (74%) and high frequency of *tetA* determinant, which also agrees with regional reports that define tetracycline and its resistance genes as widespread in animal‐associated *E. coli* populations. South Asian studies indicate the same trends in a number of studies. Studies in West Bengal and other parts of India reported increasing resistance in mastitis‐related Gram‐negative pathogens, and common resistance to previously used agents like ampicillin, tetracycline, and streptomycin [38, 39]. Current surveys in Bangladesh and the adjacent nations also report high percentages of beta‐lactam and tetracycline resistance and often carry genes of the bla and tet family, which is indicative of the pattern of high pressure of selection by the use of these drug classes in dairy practice [14, 40].

The agreement between phenotypic resistance and PCR results of the study resistance genes in this dataset has mechanistic underpinnings, which are well described. Beta‐lactam resistance linked to *blaTEM* and *blaCTX-M* is an expression of hydrolysis of the beta‐lactam ring by enzymes, thus making penicillins and most cephalosporins inefficient [41]. Horizontal transfer of commensal and pathogenic strains occurs through plasmid‐borne bla genes, which increases their transmission in farm settings [11]. Our isolates have tetracycline resistance that is highly attributed to *tetA*, an efflux gene that actively releases tetracycline out of the bacterial cytoplasm, bringing drug concentration to levels much lower than inhibitory levels. The most typical types of efflux are found in *E. coli* and are often encoded on mobile vectors, thus spreading quickly. It is also in this mechanism that the partial or intermediate susceptibility to corresponding related tetracyclines like doxycycline and minocycline can be described based on the efflux specificity [42]. Furthermore, 59.53% isolates showed phenotypic resistance to streptomycin and the presence of *aadA1* gene in 50% isolates in the present study, indicating a good correlation between the phenotypic and genotypic findings and suggesting that streptomycin resistance is prevalent in this region. MDR phenotypes of a number of isolates can be explained by the presence of several resistance genes on the plasmids or integrons. The *blaCTX-M* in some of the isolates is an issue of particular concern given that ESBLs reverse third‐generation cephalosporins, and therapeutic choices are limited [43, 44].

The abundance of *E. coli* phylogroup A1 in the isolates and a low number of *E. coli* phylogroup B2 strains give important clues to the epidemiology and pathogenic potential of mastitis‐associated *E. coli*. *E. coli* strains associated with mastitis in cattle have been shown in previous studies to be mainly represented by phylogroups A and B1, typically associated with the gastrointestinal tract and environmental reservoirs, respectively, which are not considered specialized pathogens of the mammary gland [45, 46]. The high percentage of phylogroup A1 in this study would indicate that the majority of cases of mastitis in this study were probably from the fecal and environmental contamination sources, including bedding, manure, water, and milking equipment. In contrast, the detection of phylogroup B2 isolates, although limited in frequency, is clinically significant because phylogroup B2 is strongly associated with extraintestinal pathogenic *E. coli* (ExPEC) strains that possess a greater extent of virulence‐associated genes, including adhesins, iron‐acquisition systems, toxins, invasins, and serum‐resistance factors [47, 48]. These virulence factors are important for bacterial colonization, persistence, immune evasion, and tissue invasion, contributing to their pathogenic potential. A limitation of the present study is that virulence profiling was restricted to only three major virulence genes (*eae, stx1*, and *stx2*), none of which were detected among the *E. coli* isolates. Although the absence of these genes suggests a low likelihood of the isolates belonging to classical Shiga toxin–producing or enteropathogenic *E. coli* pathotypes, it does not necessarily indicate a lack of pathogenic potential. Numerous other virulence‐associated genes involved in adhesion, iron acquisition, serum resistance, toxin production, biofilm formation, and host colonization have been reported in mastitis‐associated and extraintestinal pathogenic *E. coli* strains [49, 50]. Therefore, the limited virulence gene screening performed in this study may not fully capture the pathogenic characteristics of the isolates. Comprehensive characterization of a broader range of virulence determinants, particularly those associated with extraintestinal pathogenic *E. coli*, would provide a more complete understanding of their virulence potential, zoonotic significance, and possible transmission risk to humans through direct animal contact or the dairy production chain.

## 5. Conclusion

The study offers extensive data on the fact that *E. coli* is a major etiological agent of bovine clinical mastitis in the northern part of Bangladesh and most of the isolates are MDR. The extensive resistance to b‐lactam and tetracycline antibiotics and the abundance of *blaTEM*, *blaCTX-M*, and *tetA* gene indicate the widespread distribution of mobile genetic factors that cause antimicrobial resistance in dairy setting. Even though the majority were commensals of the A1 phylogroup, the occurrence of B2 suggests the potential risk of zoonosis and the potential spread of the resistance determinants to human‐associated bacteria. The lack of classical virulence genes (*eae*, *stx1*, *stx2*) is an indication that mastitis in this area is predominantly due to opportunistic environmental *E. coli* and not enteropathogenic ones. Nevertheless, the experimented genotypic–phenotypic concordance proves that the resistance of the isolates to the effects of selective pressure happens to be based on resistance factors rather than on virulence factors. Such results underscore the need to use antimicrobials wisely in veterinary practice, to pretest susceptibility prior to treatment, and to strictly regulate the sales of over‐the‐counter antibiotics.

## Funding

This study was supported by the Institute of Research and Training (IRT) of Hajee Mohammad Danesh Science and Technology University, Bangladesh, Grant number: HSTU/IRT/3869, 2022‐23.

## Conflicts of Interest

The authors declare no conflicts of interest.

## Data Availability

The data that support the findings of this study are available from the corresponding author upon reasonable request.
